# Small Cell/Neuroendocrine Prostate Cancer: A Rare Treatment-Resistant Variant Presenting as Acute Onset Severe Back Pain

**DOI:** 10.7759/cureus.52379

**Published:** 2024-01-16

**Authors:** Su T Khine, Sania Ajmal, Qazi Azher, Adiraj Singh

**Affiliations:** 1 Internal Medicine, Hurley Medical Center/Michigan State University, Flint, USA; 2 Internal Medicine/Pediatrics, Hurley Medical Center/Michigan State University, Flint, USA; 3 Pathology and Laboratory Medicine, Hurley Medical Center/Michigan State University, Flint, USA

**Keywords:** psa negative prostate cancer, oncology, adt (androgen deprivation therapy), bony metastases, intractable back pain, neuroendocrine prostate cancer

## Abstract

Adenocarcinoma of the prostate is the most frequent subtype of prostate cancer. Being an androgen-driven disease, androgen deprivation therapy (ADT) is one of the mainstay treatments for prostatic adenocarcinoma. ADT however can induce androgen resistance and can cause neuroendocrine differentiation of the cells and subsequently can lead to the emergence of neuroendocrine prostate cancer (NEPC). NEPC, despite being rare, is very aggressive with a very low survival period. The majority of the NEPC cases are treatment-emergent. There is no definite guideline on screening for the development of NEPC for patients who are on ADT. Our case highlights the lethality and aggressiveness of NEPC and the relationship between ADT and NEPC. More research is needed to compare different imaging techniques for early detection and identification of NEPC and to establish screening protocols for patients at risk of developing NEPC while on ADT.

## Introduction

Prostate cancer is one of the most common causes of cancer in men globally, with a reported incidence of 1.4 million in the year 2020 [1]. In the United States, prostate cancer accounts for approximately 29 percent of cancer diagnoses [2]. Adenocarcinoma is the most common type of prostate cancer. Other less common subtypes include small-cell carcinoma, transitional cell carcinoma, and neuroendocrine tumors. Neuroendocrine prostate cancer (NEPC) is a rare and aggressive subtype of prostate cancer. In most cases, NEPC has been related to androgen deprivation therapy (ADT) which is commonly used for metastatic prostate cancer and also as adjuvant therapy for high-risk localized prostate cancer. On the other hand, de novo NEPC with no previous treatment with ADT accounts for less than 2% of prostate cancer cases [3]. Chemotherapy remains the mainstay of treatment for NEPC yet the prognosis is very poor as most patients have metastatic disease at the time of diagnosis. 

We are presenting a case of stage 4 prostate cancer that has been in remission as evident with negative prostate-specific antigen (PSA) and normal bone scan two months prior and now presented with acute severe back pain which was subsequently diagnosed as neuroendocrine prostate cancer with metastases to the whole spine.

## Case presentation

The patient is a gentleman in his 60s who presented to the emergency department with back pain for one day. He has a past medical history significant for bladder cancer status post transurethral resection, stage 4 prostate cancer with Gleason stage 4+3 diagnosed three years prior. A year ago, the patient was started on leuprolide and apalutamide for a high PSA of 154 ng/ml. PSA levels declined steadily and at the office follow-up two months prior to presentation, the PSA level was normal and the bone scan was negative. 

**Figure 1:**
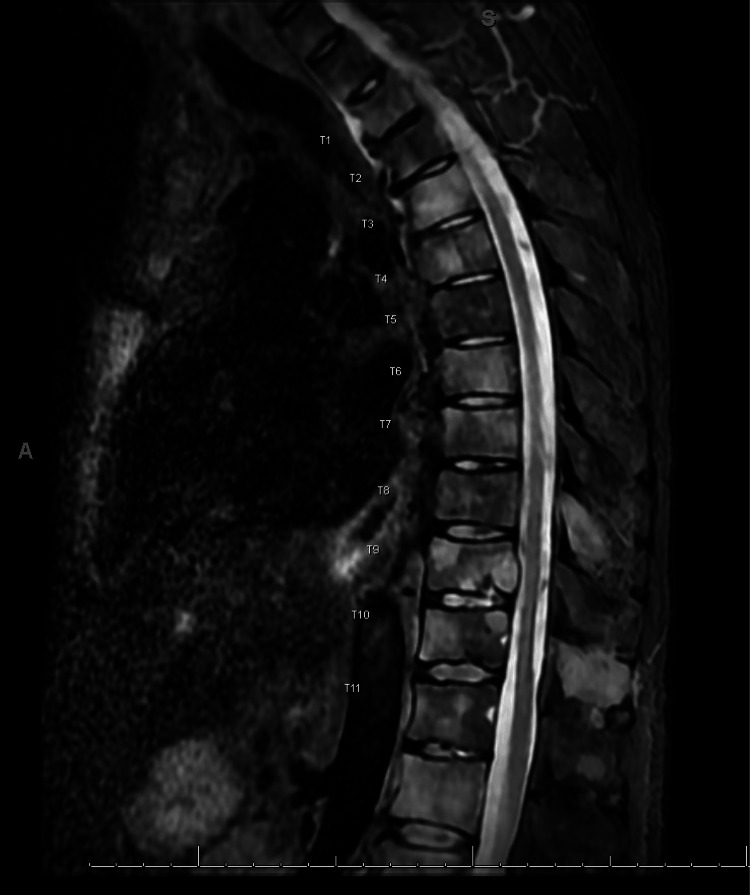
MRI T spine sagittal view with contrast showing extensive abnormal enhancement throughout the osseous structures compatible with metastases

The patient now presented with concerns of severe pain in the upper back between the shoulder blades for a few hours without any history of trauma or inciting events. He reported the pain to be radiating down his spine without any motor or sensory deficits in the lower extremities or perineal region. He was initially worked up for aortic dissection and computerized tomography (CT) of the aorta was negative. Laboratory studies are unremarkable except for anemia with a hemoglobin of 11 g/dl. Due to severe intractable back pain which did not respond to a few doses of intravenous pain medications, magnetic resonance imaging (MRI) of the thoracic spine was performed. MRI revealed extensive metastatic lesions throughout the entire thoracic spine including the sternum and bilateral ribs with pathologic compression fracture at T1 and osseous metastases with cortical breakthrough at T9 level resulting in moderate to severe canal stenosis at T8-9 (Figure 1).

**Figure 2:**
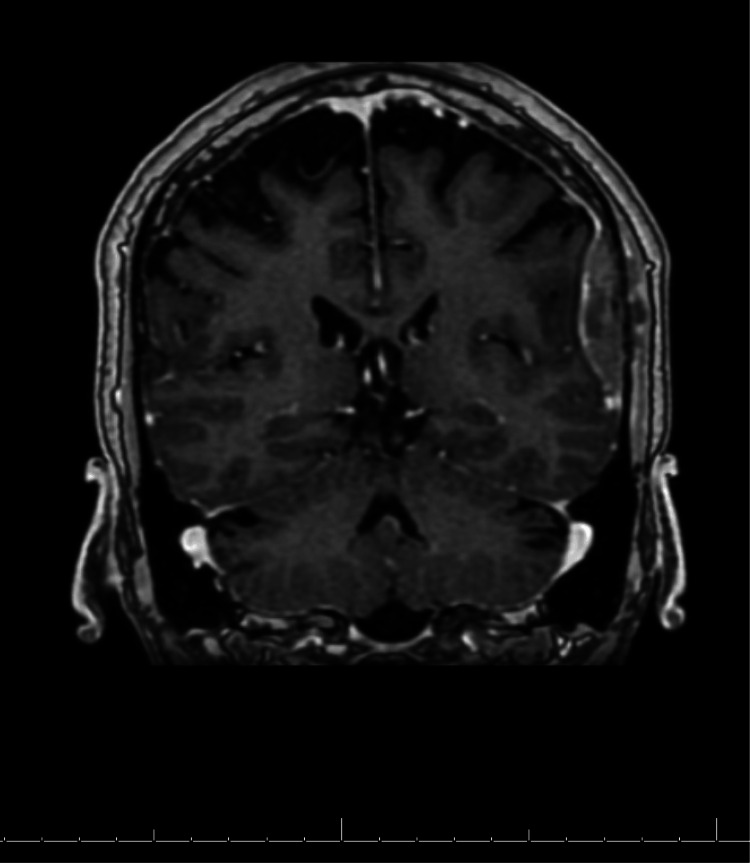
MRI brain, coronal view, showing dural-based lesions in the mid posterior left temporal lobe measuring 1.2 x 4.5 cm

Neurosurgery and oncology were consulted. MRI of the cervical and lumbar spine as well as the head further revealed widespread metastatic disease in the cervical and lumbar spine, calvarium, and dural-based metastatic lesions at the temporal lobe (Figure 2).

The patient was started on dexamethasone 8 mg twice a day along with pain management. PSA was repeated and came back as normal. Neurosurgery did not recommend any acute intervention. Interventional radiology was consulted and the patient underwent CT-guided bone biopsy of L1 vertebra with kyphoplasty. Radiation oncology was consulted and the patient was started on palliative radiation. During the hospital stay, back pain was progressing and was difficult to control. The patient required high doses of morphine and hydromorphone and ultimately started on fentanyl patches which controlled the pain. The patient was discharged after adequate pain control was achieved. 

**Figure 3:**
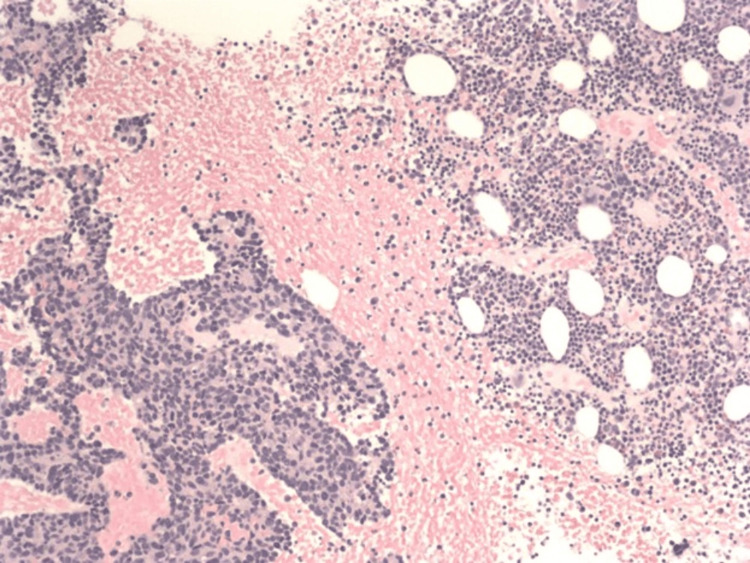
Low-power hematoxylin and eosin (H&E) stained slide at 100x showing cohesive elements indicative of metastatic tissue, note high nuclear/cytoplasmic ratio in the cells

**Figure 4:**
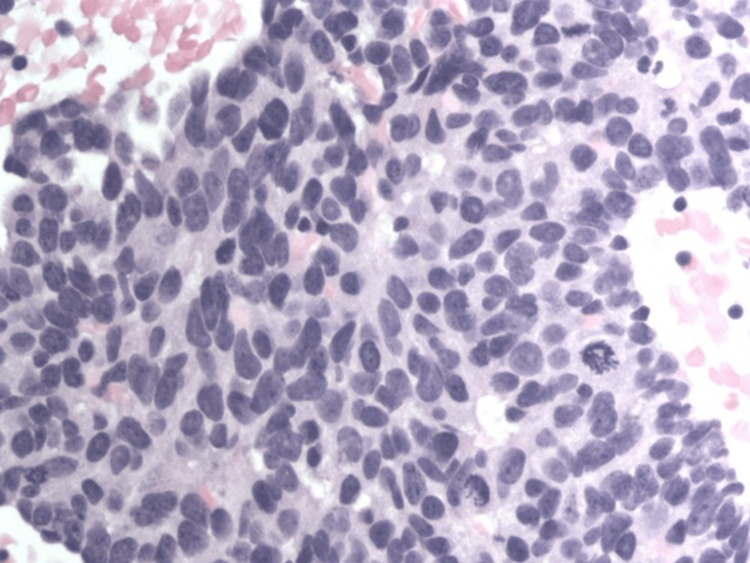
High-power H&E-stained slide at 400x showing an increased nuclear-cytoplasmic ratio; some mitotic figures seen along with salt and pepper chromatin

**Figure 5:**
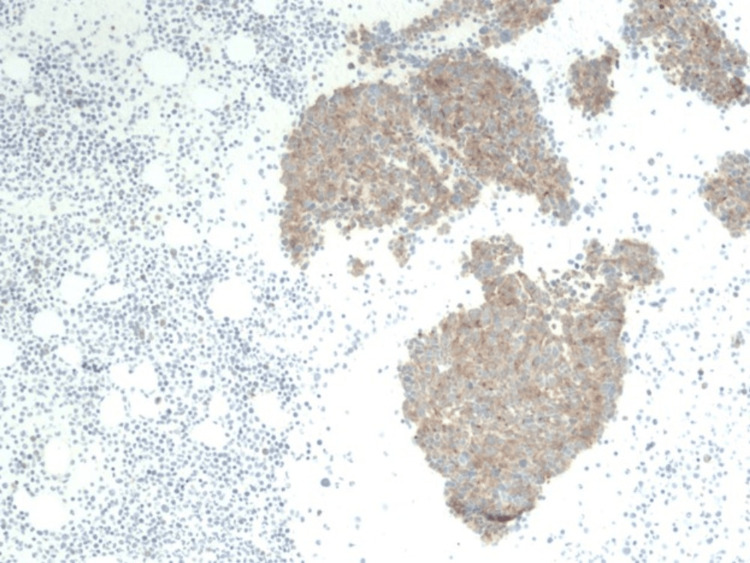
Lower power slide at 100x showing synaptophysin staining, which is a tumor marker for neuroendocrine tumors

**Figure 6:**
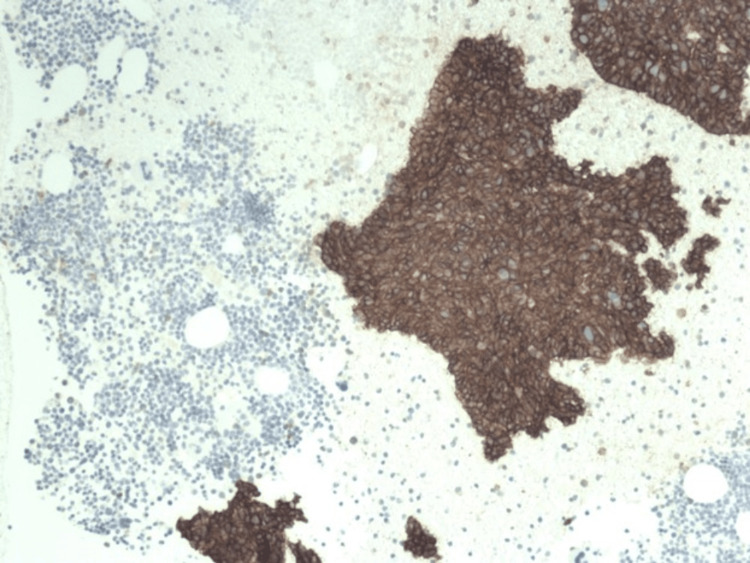
Microscopic findings of a neuroendocrine tumor Immunohistochemical staining for CD 56 (Low power view at 100x) showing positivity for tumor cells in cytoplasmic pattern. The staining is not seen in marrow cells, demarcating the metastatic tissue and the normal tissue

Biopsy results later came back as high-grade metastatic neuroendocrine carcinoma consistent with small cell/neuroendocrine prostate carcinoma (trans-differentiated cancer) (Figures 3-6).

The patient was started on etoposide and cisplatin but was again admitted one month later due to uncontrolled pain. The patient continued to have worsening back pain with enlarging tender nodules in his scalp. He also started developing some neurological symptoms such as numbness, tingling, and weakness in his right leg. Systemic therapy was discontinued due to the lack of response. The patient was later transitioned to hospice care. 

## Discussion

Adenocarcinoma is the most common type of prostate cancer, accounting for more than 95% of the cases. Since it is an androgen-driven disease, ADT has been widely used especially in locally advanced or metastatic disease. However, ADT can also induce resistance to androgen signaling and lead to the emergence of NEPC, a rare but aggressive subtype of prostate cancer that expresses neuroendocrine markers and does not depend on androgen for growth. Recent studies have reported an expected increasing incidence of NEPC in the near future due to the more widespread use of ADT [3]. Even though the incidence is low, with expected increasing cases, physicians should be aware of the potential for neuroendocrine differentiation in prostate cancer patients receiving ADT. 

ADT is one of the mainstay treatments in prostate cancer that has shown survival benefits in some studies [4,5]. PSA is a marker frequently used to monitor the therapeutic response to ADT as well as disease progression. Imaging studies such as bone scans and positron emission tomography scans are indicated only when PSA levels are elevated or when the patients have specific symptoms. However, these recommendations are mainly for monitoring disease progression or remission only in cases of adenocarcinoma of the prostate. There is no definite guideline on how to monitor the development of neuroendocrine differentiation, especially in patients receiving ADT. In addition, NEPC is PSA-independent and there is limited data on using conventional modalities to detect NEPC. In our case, the patient has a good therapeutic response to ADT as evident with normal PSA and negative bone scan two months prior to diagnosis of widespread metastatic NEPC. It brings up the question of whether bone scans can be used for the detection of NEPC if suspected. NEPC is a very aggressive disease with a median survival period of only seven months. Most cases already have widespread metastases at the time of diagnosis which partly contributed to the high mortality [3]. Early detection would be beneficial in clinical management. There are a few studies indicating the possible use of chromogranin A and neuron-specific enolase to detect neuroendocrine cells. Some data reflect that once ADT is started, monitoring the level of these markers can inform us in terms of a patient's prognosis and potential of the prostate cancer to acquire resistance to ADT and thereby convert to a neuroendocrine tumor. Elevated levels of chromogranin raise strong suspicion for neuroendocrine conversion of prostate cancer in the setting of antiandrogen therapy, which can potentially be used as a tool in patients undergoing antiandrogen therapy [6-8]. More studies are needed to identify reliable biomarkers and imaging modalities for NEPC as well as to establish screening protocols for patients at risk of developing NEPC while on ADT.

## Conclusions

Owing to the advancement in medical therapies, ADT is more widely used and the incidence of treatment-emergent NEPC is expected to be increasing in the near future. NEPC is a very aggressive cancer with no correlation to levels of PSA and the majority of cases are already in the metastatic stage at the time of diagnosis yet have normal PSA. Studies show that this conversion occurs at the cellular level as the neuroendocrine cells do not have androgenic receptors, thus failing the ADT treatment. At this point, tracking markers more sensitive to neuroendocrine cells can give more information about patient's prognosis or possible conversion. Research studies are required to improve guidelines on screening patients on ADT for NEPC. Physicians should have a high degree of suspicion for the development of NEPC in patients who present with atypical back pain and have been on ADT. 
